# Supplementary material for: Analysis of the Predictors and Consequential Factors of Emotional Exhaustion Among Social Workers: A Systematic Review
Source: Healthcare (Basel). 2025 Mar 4;13(5):552. doi: 10.3390/healthcare13050552 (PMC11899651; doi:10.3390/healthcare13050552)
Supplement: Supplementary file 1 [file healthcare-13-00552-s001.zip › Table S2 complete.pdf]

Table S2. Methodological quality level according to the CRF-QS items [62].

Article	CRF-QS items																			
	**1**	**2**	**3**	**4**	**5**	**6**	**7**	**8**	**9**	**10**	**11**	**12**	**13**	**14**	**15**	**16**	**17**	**18**	**19**	**Total**
[67]	1	1	1	1	1	1	1	1	1	1	0	0	1	1	0	1	1	0	1	15
[68]	1	1	1	1	1	1	1	1	1	1	0	0	1	1	0	1	1	1	1	16
[69]	0	1	1	1	1	0	1	1	1	1	0	0	1	1	0	1	1	0	0	12
[70]	1	1	1	1	1	0	1	1	1	1	0	0	1	1	0	1	1	1	1	15
[71]	1	1	1	1	1	0	1	1	1	1	0	0	1	1	0	1	1	1	1	15
[72]	1	1	1	1	1	0	0	1	1	1	0	0	1	1	0	1	1	1	0	13
[73]	1	1	1	1	1	1	1	1	1	1	0	0	1	1	0	1	1	1	1	16
[74]	1	1	1	1	1	1	1	1	1	1	0	0	1	1	0	1	1	1	1	16
[75]	1	1	1	1	1	1	0	1	1	1	0	0	1	1	0	1	0	0	0	12
[76]	1	1	1	1	1	0	1	1	1	1	0	0	1	1	1	1	1	1	1	16
[77]	0	1	1	1	1	1	1	1	1	1	0	0	1	1	0	1	1	0	1	14
[78]	0	1	1	1	1	1	1	1	1	1	0	0	1	1	0	1	1	1	1	15
[79]	1	1	1	1	1	1	1	1	1	1	0	0	1	1	0	1	1	1	1	16
[80]	1	1	1	1	1	1	1	1	1	1	0	0	1	1	0	1	1	0	1	15
[81]	1	1	1	1	1	0	1	1	1	1	0	0	1	1	0	1	1	1	1	15
[82]	1	1	1	1	1	1	1	1	1	1	0	0	1	1	0	1	1	0	0	14
[83]	1	1	1	1	1	1	1	1	1	1	0	0	1	1	0	1	1	1	1	16
[84]	0	1	1	1	1	1	0	1	1	1	0	0	1	1	0	1	1	1	0	13
[85]	1	1	1	1	1	0	1	1	1	1	0	0	1	1	0	1	1	0	0	13
[86]	1	1	1	1	1	1	1	1	1	1	0	0	1	1	0	1	1	0	0	14
[87]	1	1	1	1	1	1	1	1	1	1	0	0	1	1	0	1	1	1	1	16

Note: Maximum score of 19: poor quality level (⩽11 points), acceptable (between 12 and 13 points), good (14 and 15 points), very good (16 and 17 points) and excellent (18 and 19 points).
